# Ergosterol extraction: a comparison of methodologies

**DOI:** 10.1099/acmi.0.000490.v4

**Published:** 2023-04-28

**Authors:** Thomas I. Wilkes

**Affiliations:** ^1^​ School of Life and Medical Sciences, College Lane Campus, University of Hertfordshire, Hatfield, Hertfordshire, AL10 9AB, UK; ^2^​ School of Water, Energy, and Environment, Cranfield University, Bedford, Bedfordshire, MK43 0AL, UK

**Keywords:** chloroform, cyclohexane, ergosterol, fungal biomass, methanol, mycorrhizal fungi

## Abstract

Ergosterol is a component of the cell membrane of mycorrhizal fungi and is frequently used to quantify their biomass. Arbuscular mycorrhizal (AM) fungi and ectomycorrhizal (ECM) fungi establish a symbiotic relationship with a respective host plant. Several methods are currently employed for quantification of ergosterol; however, these utilise a series of potentially hazardous chemicals with varying exposure times to the user. The present comparative study aims to ascertain the most reliable method to extract ergosterol whilst limiting hazard exposure to the user. Chloroform, cyclohexane, methanol and methanol hydroxide extraction protocols were applied to a total of 300 samples of root samples and a further 300 growth substrate samples across all protocols. Extracts were analysed via HPLC methodologies. Chromagraphic analysis showed chloroform-based extraction procedures produced a consistently higher concentration of ergosterol in both root and growth substrate samples. Methanol hydroxide, without the addition of cyclohexane, produced a very low concentration of ergosterol, with a reduction of quantified ergosterol of between 80 and 92 % compared to chloroform extractions. Hazard exposure was greatly reduced following the chloroform extraction protocol when compared with other extraction procedures.

## Data Summary

All generated data are provided within the paper.

**Impact Statement**

Ergosterol extractions typically employ methanol hydroxide as one of the main solvents. Inflated ergosterol quantification, and fungal biomass via equation transformation, can result from corrosive damage to plant cells. Therefore, alternative methods must be investigated. Furthermore, user exposure to hazardous chemicals, as well as overall length of the procedure, is greater than other solvent-based ergosterol extraction procedures carried out in the present study. Chloroform ergosterol extraction was seen to require the shortest period of user exposure to hazardous chemicals as well as the shortest extraction processing time. Chloroform extractions also were able to produce a greater quantity of ergosterol from the same samples as those subjected to methanol hydroxide extraction. This may suggest a chemical reaction between methanol hydroxide and ergosterol that reduces the quantity of the compound able to be extracted.

## Introduction

Both arbuscular mycorrhizal (AM) and ectomycorrhizal (ECM) fungi form close symbiotic relationships with a host plant, and aid in the acquisition of soil nutrients provided to their host [1]. AM fungi have been studied in relation to agricultural crop growth, development and yield for a wide range of crops produced worldwide, whereas ECM fungi have been studied in woodland and forestry ecology for reforestation and agroforestry. Both AM and ECM fungi, however, are able to aid in the sequestration of carbon by increasing their requirement for photosynthetically produced carbohydrates [2–4]. Both types of mycorrhiza increase soil quality, but can be influenced positively or negatively from an applied method of land management [5–7]. To determine the influence of land management on the abundance and biomass of mycorrhizal fungi, ergosterol can be extracted from a soil and/or root sample to indicate biomass along with other biomarkers as proxy indicators [7–11].

Ergosterol (ergosta-5,7,22-trien-3β-ol: C_28_H_44_O) is a phytosterol consisting of three double bonded carbon atoms throughout the molecule and a beta-hydroxyl group [12]. Found within fungal cell membranes, ergosterol aids in the fluidity of the cell membrane and allows for the continued transport of physiological metabolites across the growth temperature of the fungal organism [8]. With similarities to cholesterol in animal cell membranes, ergosterol’s hydroxyl group interacts with water molecules in a similar way that heads of phospholipids interact with water molecules internally and externally within an animal cell [13]. The main structure of the ergosterol sterol is embedded within the phospholipid membrane with the fatty acid chains, encompassing the hydrophobic elements of the molecule [14].

Current ergosterol extraction procedures typically employ the use of cyclohexane as an organic solvent along with alkaline methanol [9–11, 15, 16]. The use of cyclohexane requires additional safety measures to be implemented and exposes the user to long-term health hazards [17]. Other extraction methods, with the absence of cyclohexane, utilize methanol and alkaline methanol – potassium hydroxide and methanol [methanol hydroxide (MeOH)] – for varying lengths of extraction time [10, 15, 16].

The present study aims to compare different ergosterol extraction methods and comment on the degree of chemical safety each method presents to the user as well as the quantity of ergosterol extracted for reliable fungal biomass estimation. Ergosterol is analysed here via HPLC methodology.

## Methods

Plant growth of two ECM fungi-supporting species [English oak – *Quercus robur* (*n*=20), and roses – *Rosa gallica* (*n*=20)] and two AM fungi-supporting species [wheat – *Triticum aestivum* (*n*=20), oats – *Avena sativa* (*n*=20)] were grown under controlled conditions (20±2°C, 18±5 % humidity, 15 500 lumens) and grown in 50 % perlite and 50 % vermiculite as growth substrate with the addition of 50 g J Arthur Bowers multipurpose compost as a mycorrhizal inoculum. Watering was carried out once per week to a total volume of 100 ml, along with a source of liquid nutrient (BabyBio) applied to each plant every 4 weeks diluted as per the manufacturer’s instructions. Plants were sampled at 6 months post-germination where a maximum mass of 15 g of growth substrate (*n*=300 per ergosterol extraction protocol per plant species) and 1 g of root tissue (*n*=300 per ergosterol extraction protocol per plant species) was taken and stored at −20 °C until ergosterol extraction could be performed.

### Sample pre-treatment

A total of 2 g sampled rhizosphere growth substrate and 1 g root material was air dried at 25 °C for 48 h. Samples were determined to be dried after three consistent and consecutive weight measurements a minimum of 6 hs apart.

### Ergosterol extraction

#### Non-alkaline (methanol) extraction protocol

**Fig. 1.**
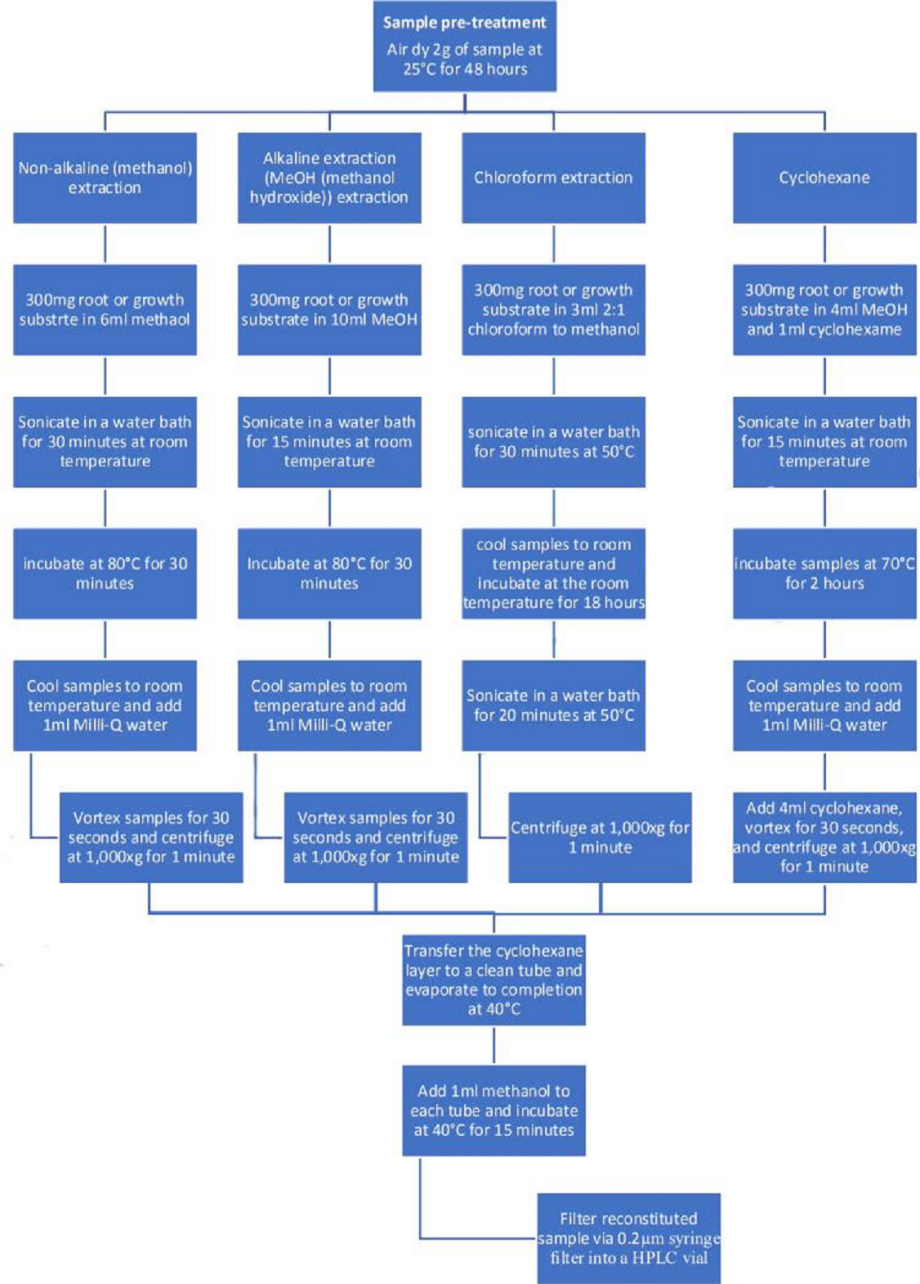
Flowchart summary of the four ergosterol extraction procedures.

A modified method of Millie-Lindblom *et al.* [9] was used for ergosterol extraction via methanol, as summarized in Fig. 1. Of both growth substrate and root samples, 300 mg was weighed into 50 ml centrifuge tubes. To each sample, 6 ml HPLC-grade methanol was added and sonicated in an ultrasonic water bath for 30 min before incubation at 80 °C for a maximum of 30 min. Samples were allowed to cool to room temperature and 1 ml of Milli-Q water was added, then vortexed at maximum speed for 1 min. Samples were centrifuged at 1 000 ***g*** for 1 min. The methanol layer was transferred to a clean tube and heated continuously in a 40 °C water bath until methanol had evaporated to completion. To each tube, 1 ml of HPLC-grade methanol was added and incubated at 40 °C for 15 min then filtered through 0.2 µm nylon membrane syringe filters (Chromatography Direct) into amber glass HPLC vials for later analysis. All chemicals and reagents were purchased from Thermo Fisher Scientific.

#### Alkaline extraction (MeOH) protocol

A modified method of Caroll [15] was used for ergosterol extraction via MeOH. Of both growth substrate and root samples, 300 mg was weighed into 50 ml centrifuge tubes, as summarized in Fig. 1. Potassium hydroxide was added to HPLC-grade methanol until 10 % (w/v) was achieved. To each centrifuge tube, 10 ml of KOH in methanol was added and sonicated in an ultrasonic water bath for 15 min before incubation at 80 °C for a maximum of 30 min. Samples were allowed to cool to room temperature and 1 ml of Milli-Q water was added, then vortexed at maximum speed for 1 min. Samples were centrifuged at 1 000 ***g*** for 1 min. The methanol layer was transferred to a clean tube and heated continuously in a 40 °C water bath until methanol had evaporated to completion. To each tube, 1 ml of HPLC-grade methanol was added and incubated at 40 °C for 15 min then filtered through 0.2 µm nylon membrane syringe filters (Chromatography Direct) into amber glass HPLC vials for later analysis. All chemicals and reagents were purchased from Thermo Fisher Scientific.

#### Chloroform extraction protocol

A modified method of Alekseyeva *et al*. [18] was used for ergosterol extraction via methanol and chloroform. Of both growth substrate and root samples, 300 mg was weighed into 50 ml centrifuge tubes, as summarized in Fig. 1. To each sample, 3 ml of 2 : 1 chloroform to methanol was added and sonicated for 30 min at 50 °C in a closed tube. Samples were then allowed to cool to room temperature, followed by incubation at room temperature for 18 h. Samples were subsequently sonicated at 50 °C for 20 min and centrifuged at 1000 ***g*** for 1 min. Supernatant was transferred to a clean tube and heated continuously in a 40 °C water bath until methanol and chloroform had evaporated to completion. To each tube, 1 ml of HPLC-grade methanol was added and incubated at 40 °C for 15 min then filtered through 0.2 µm nylon membrane syringe filters (Chromatography Direct) into amber glass HPLC vials for later analysis. All chemicals and reagents were purchased from Thermo Fisher Scientific.

#### Cyclohexane extraction protocol

A modified methodology originally developed by Millie-Lindblom *et al*. [9] was employed for the extraction of ergosterol [7]. Of both growth substrate and root samples, 300 mg was weighed into 50 ml centrifuge tubes, as summarized in Fig. 1. Potassium hydroxide was added to HPLC-grade methanol until 10 % (w/v) was achieved. To each centrifuge tube, 4 ml KOH in methanol and 1 ml cyclohexane was added and sonicated in an ultrasonic water bath for 15 min before incubation at 70 °C for a maximum of 2 h. Samples were cooled to room temperature and 1 ml of Milli-Q water was added with a further 4 ml cyclohexane, vortexed at maximum speed for 60 s then centrifuged at 1000 ***g*** for 60 s. The cyclohexane fraction was transferred to a clean test tube and all cyclohexane as evaporated, before 1 ml of HPLC-grade methanol was added and each tube incubated at 40 °C for 15 min then filtered through 0.2 µm nylon membrane syringe filters (Chromatography Direct) into amber glass HPLC vials for later analysis. All chemicals and reagents were purchased from Thermo Fisher Scientific.

### Monitoring volatile exposure to the user

A ToxiRAE Pro PID (Honeywell) was kept by the user throughout the extraction procedure to monitor volatile organic compounds (VOCs) within the localized atmosphere to the user.

### HPLC running protocol

The HPLC protocols were performed as described by Wilkes *et al*. [7], as a modified methodology of Mille-Lindblom *et al*. [9].

### Fungal biomass estimation

Fungal biomass was determined from measured ergosterol concentration according to equation 1 [19]:

Fungal biomass (FB)(μg/g) = Ergosterol (μg/g)×f ×Rf

where *f* is 250 and *Rf* (the recovery factor) is 1.61.

### Confirmation of AM fungi

Wheat and oak root sections were stained following the procedure of Wilkes *et al*. [20] for the visual confirmation of root intracellular AM fungal root structures.

### Statistical analysis

Multivariate ANOVAs were used to determine if significant differences between sample/substrate type, plant species and ergosterol extraction produced an overall impact on the quantification of ergosterol. Single factor ANOVAs were performed between plant species of the same ergosterol extraction protocol, with post-hoc t testing, in order to determine the most effective ergosterol extraction procedure. Further t testing was caried out between ergosterol extraction protocols of the same plant species. Comparisons between ergosterol quantification of ECM and AM fungi were made via t testing. All data were analysed via R (version 4.1.0).

## Results

**Fig. 2.**
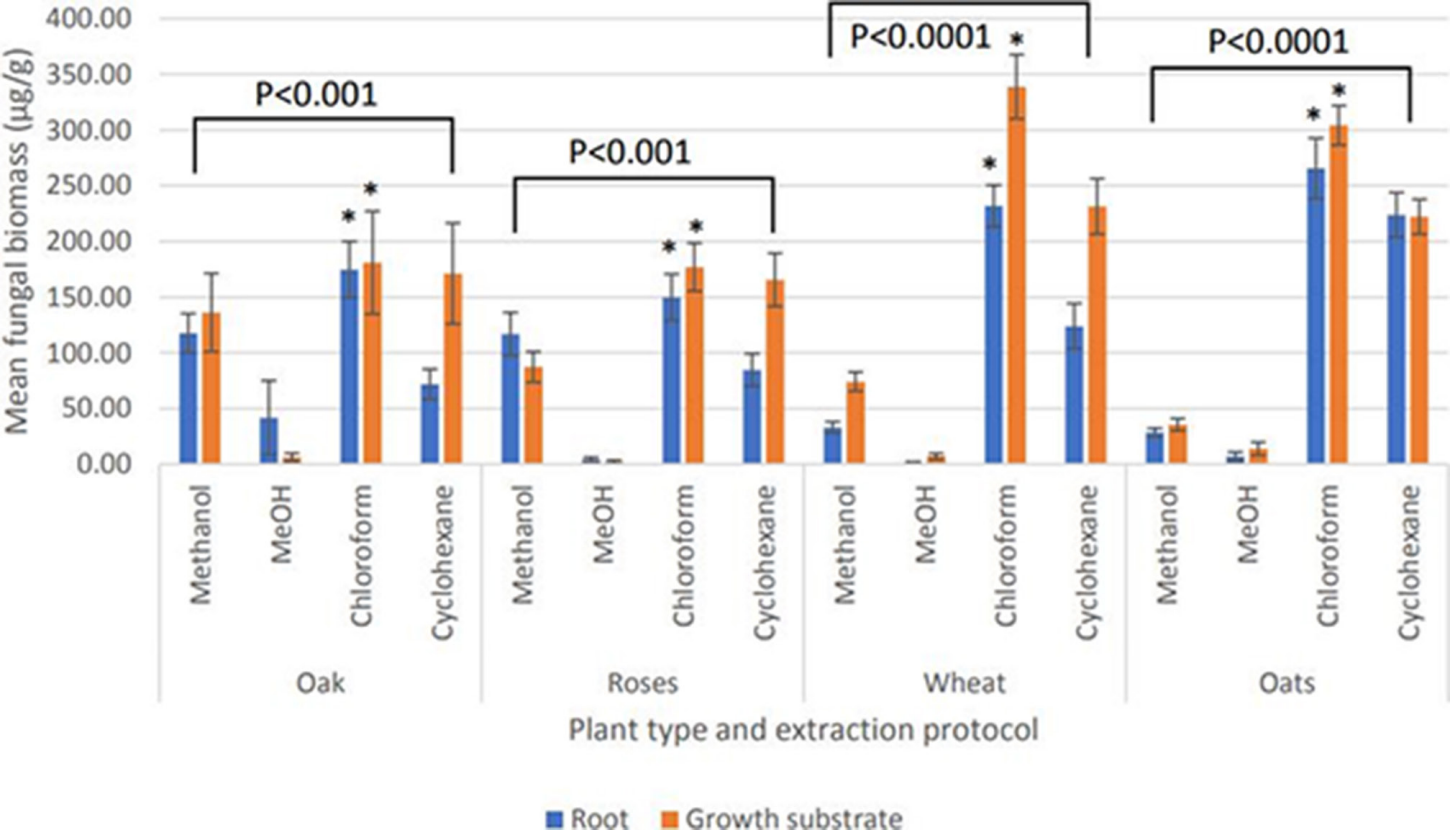
Mean fungal biomass (*n*=300 root samples overall, *n*=3000 growth substrate samples overall) of extracted plant root and rhizosphere growth substrate samples from controlled glasshouse growth of four developing plants (oak – *Quercus robur*, roses – *Rosa gallica*, wheat – *Triticum aestivum*, oats – *Avena sativa*) at 6 months from germination, between four methods of ergosterol extraction. *A significant increase in quantified ergosterol quantification from a post-hoc t test (*P*<0.00001) of the ANOVA (presented *P* values) across all extraction protocols for both root and growth substrate samples within the same plant species. Error bars are constructed from the standard error of the mean.

The method used for the extraction of ergosterol was noted to have a large significance in the overall concentration of extractant and subsequent quantification of fungal biomass [*P*<0.0001, degrees of freedom (d.f.): 3,3996, F value: 5.99, F critical: 2.69, multi-factor ANOVA]. Post hoc t testing further revealed that MeOH had a significantly negative impact on ergosterol extraction (*P*<0.002, d.f.: 245, t.stat: 2.01, paired equal variance t test) reducing the overall quantity extracted, whilst chloroform had a significantly increased impact on the extraction of ergosterol (*P*<0.0001, d.f.: 245, t.stat: −1.25, paired equal variance t test) (Fig. 2).

**Fig. 3.**
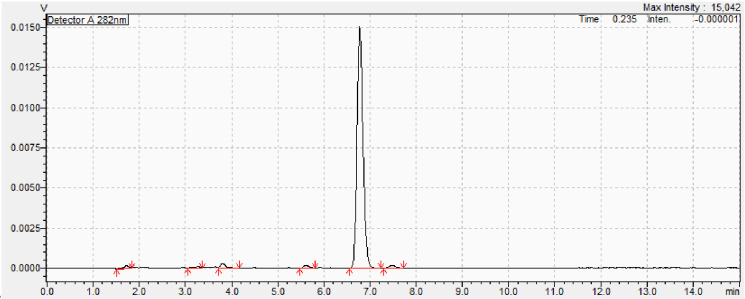
Ergosterol standard run following the HPLC protocol of Wilkes *et al*. [7] and Millie-Lindblom *et al*. [9], indicating an ergosterol retention time of 6.8 min.

**Fig. 4.**
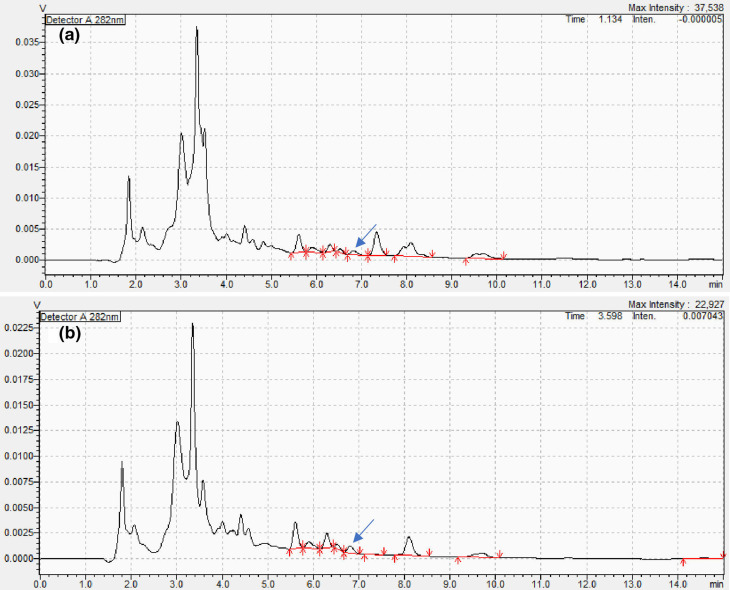
HPLC chromatograms of (**a**) root samples of English oak (*Quercus robur*) and (**b**) growth substrate of *Q. robur* sampled 6 months post-germination. Ergosterol was detected at 6.8 min (indicated by arrow) from comparison with a known ergosterol standard.

Construction of an ergosterol standard curve, via HPLC, indicated a retention time of 6.8 min (Fig. 3). Comparative analysis with root and growth substrate HPLC chromatographs (Fig. 4) presented ergosterol fractions of each extracted sample.

The total duration of the procedure is given in Table 1 along with the duration of exposure to hazardous chemicals for the respective extraction protocol. Use of a chloroform extraction protocol indicated significantly reduced hazard exposure time (*P*=0.005, d.f.: 2,9, F value: 4.04, F critical: 4.25, single-factor ANOVA). Due to the flow rate of the fume cupboard being used for all extraction procedures, monitoring of localized volatile hazards was consistently recorded at 0 ppm.

**Table 1.** User exposure time to chemical hazards associated with their respective extraction protocol for a total of 300 samples for root and growth substrate across extraction procedures and plant species

Protocol	User exposure time (min)	Incubation time (min)	Total duration (min)
Non-alkaline (methanol)	60	1080	1140
Alkaline (methanol hydroxide)	180	4320	4500
Methanol and chloroform	20	1080	1100
Cyclohexane and methanol hydroxide	180	2880	3060

## Discussion and conclusion

The present comparison of methodologies has been able to show a large range in quantified ergosterol from the same samples by using four extraction procedures. Method comparisons indicated a consistently greater degree of extracted ergosterol, proportional to fungal biomass via equation 1, via a chloroform protocol compared to the other three extraction procedures used. MeOH extractions were seen to produce consistently low fungal biomass (Fig. 2) regardless of plant species, associated mycorrhiza or sample type.

**Fig. 5.**
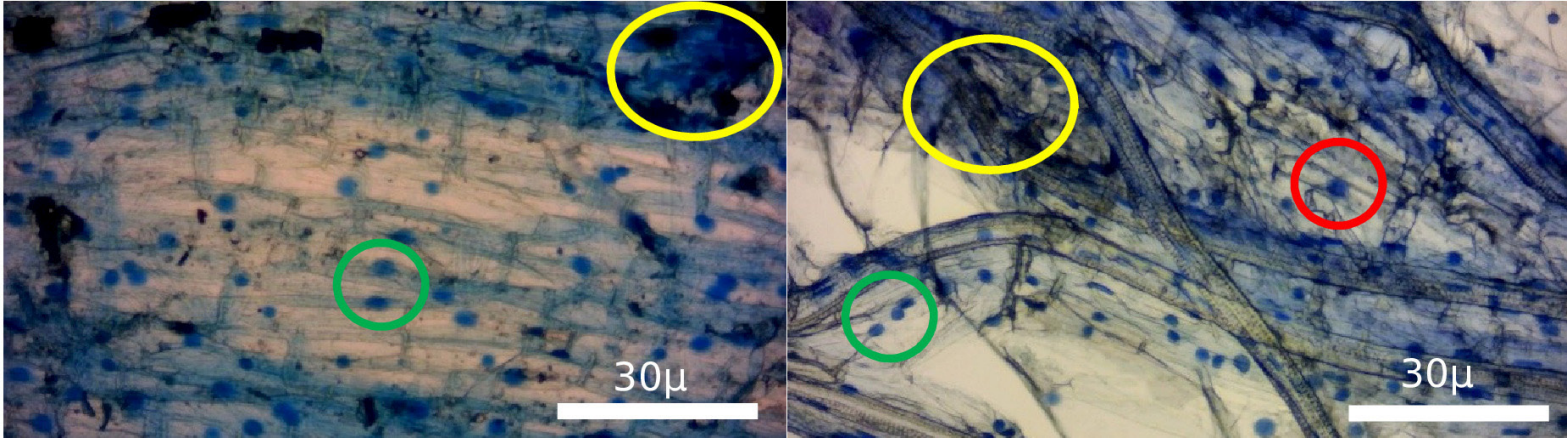
Stained wheat and oak root sections following the procedure of Wilkes *et al*. [20] for confirmation of arbuscular mycorrhizal (AM) fungi within root samples used for ergosterol extraction. Red circle: arbuscule, green circle: vesicle, yellow circle: debris.

Fungal biomass, shown in Fig. 2, shows that wheat and oats maintain a higher fungal biomass on average, regardless of extraction protocol, compared to oak and roses. As explored by Wilkes [11] the use of MeOH in a cyclohexane ergosterol extraction procedure increases the quantity of ergosterol extracted from host root samples by damaging root cortical cells and exposing intracellular fungal membranes to the extraction procedure. ECM fungal biomass extracted from host roots can be seen from oak and roses in Fig. 2 and these showed reduced fungal biomass when compared with AM fungi-hosting wheat and oats. Whilst ECM fungi also have fungal mass within root systems of their host, in the form of intercellular hyphae forming the Hartig network [21], this was not seen to increase calculated fungal biomass (Fig. 2). A further potential explanation for the difference in quantified fungal biomass is the fibrous nature of oak and roses root tissue preventing ergosterol extraction from intercellular hyphae. This is not present to the same degree within wheat and oats as both of these crop types have very malleable root tissues that can be easily stained, dissected and used for extraction procedures without the need to add a further step to remove lignin in fibrous root tissues. However, equation 1 from Montgomery *et al*. [19] utilizes an ergosterol to fungal biomass conversion ratio. Whilst such a conversion can aid calculated percentage mass of fungi in a sample, sampled soils for example, the conversion factor assumes a constant ratio between ergosterol and the mass of fungal mycelia. This is unlikely to be constant between samples. It is for this reason that, especially in the study of mycorrhizal fungi, that a single quantified parameter is not sufficient to confirm the presence of the attribute being quantified. This is the justification for staining root samples, showing AM fungal root cortical structures, in Fig. 5. Montgomery *et al*. [19] reports the typical usage of ergosterol extraction for aquatic and ectomycorrhizal fungi. It is interesting to note that the quantified ergosterol in wheat and oat samples, both root and growth substrate, indicated high concentrations of ergosterol. However, as shown by Hart and Reader [22], AM fungi do not typically contain ergosterol. Therefore, it is likely that the quantified ergosterol present in wheat and oat samples is derived from saprophytic fungi.

The use of methanol alone for the extraction of ergosterol has been utilized by several investigations with a range of success [18, 23, 24]. The main difference in methanol alone to extract ergosterol is the duration of a heat treatment in a water bath or heating block dependent on the overall volume of the sample. Verma *et al*. [25] placed samples in methanol in an 85 °C water bath for 30 min with hand shaking after 15 min followed by cooling to room temperature before filtering via a 0.22 µm syringe filter. This is similar to the methanol (non-alkaline) extraction procedure used in the present study, although the present study incubated the samples at 80 °C for 30 min with 30 min in an ultrasonic water bath. Verma *et al*. [25] found that a non-alkaline extraction procedure did not produce a greater concentration of ergosterol compared to alkaline methanol hydroxide protocols, although they did acknowledge that MeOH extractions did not allow finer soil matrices to be filtered out effectively and caused unreliable chromatographs upon HPLC analysis. This is not substantiated by the present study as an alkaline extraction procedure was able to produce the same chromatographic peak for ergosterol as methanol alone.

Typically, MeOH ergosterol extractions are coupled with cyclohexane to increase the concentration of the extracted ergosterol as seen in Fig. 2. Caroll [15] presents an MeOH ergosterol extraction without the addition of further solvents. The data presented by Caroll suggest ergosterol recovery rates ranged between 44 and 79 % for leaf samples. Such large ranges reduced the reliability of MeOH-extracted ergosterol values. From Fig. 2 of the present study, MeOH ergosterol recovery rates ranged between 0.6 and 24 % from plant root samples and between 1 and 75 % from growth substrate samples. Root samples support a greater abundance of fungal biomass compared to leaf samples. Leaf samples used by Caroll did not present with infection [15], a state that would have increased fungal biomass. The inflated values of ergosterol extracted by Caroll may be due to their standard curve being constructed from the peak height of HPLC chromatographs rather than peak area, which is proportional to the relative abundance of a corresponding molecule.

The employed chloroform/methanol ergosterol extraction procedure has several advantages over the other methods ued, including a single reaction tube, reduced equipment requirement, small volumes of solvent, and comparatively shorter extraction times (Table 1) compared to other extraction procedures. However, the majority of the extraction time in a chloroform/methanol extraction protocol comes from the incubation period where the user is not present, adding to the safety and hazard reduction benefit from such a methodology. Due to the simplicity of extraction, the chloroform/methanol extraction procedure has the potential to be used for large numbers of samples in a short period of time. Furthermore, the chemical nature of the solvents allows flexibility to extract ergosterol from a range of environmental samples, and should be tested further in continued study. Bligh and Dyer [26] and Alekseyeva *et al*. [18] provide details regarding the chemical processes around the functionality of the chloroform/methanol extraction of ergosterol. A biphasic layer is formed upon extraction, with lipids contained in the chloroform layer and non-lipid compounds within the Milli-Q water aqueous layer. Alekseyeva *et al*. [18] experimentally showed the presence of 98 % of the total extracted ergosterol was present within the chloroform layer. Both the present study and Alekseyeva *et al*. [18] dried the extracts upon completion before reconstituting in methanol.

It is acknowledged that all chemicals employed in the extraction of ergosterol are highly hazardous and pose a risk to the user, such as being corrosive and/or flammable. All extractions were carried out in a fume cupboard to reduce exposure to the user, as well as keeping a ToxiRAE Pro PID near the user to monitor volatile vapours released from the procedure. This was aided by keeping the lowest required volumes of each extractant chemical within the workspace of the fume cupboard. Such hazards, however, have to be balanced by the degree of reliably in extracting ergosterol from the sample. To this effect, the present comparison of methods would suggest the use of a chloroform extraction procedure for both root and growth substrate samples as this is also the procedure minimizing the overall duration of potential exposure to the user (Table 1). It is noteworthy that soils were not used to support the growth and development of the plant species used under controlled conditions. Therefore, the described procedures must be repeated on a wider range of sample types in order to broaden the application of the results demonstrated in the present study.
